# CEUS cardiac exam protocols International Contrast Ultrasound Society (ICUS) recommendations

**DOI:** 10.1186/s44156-022-00008-3

**Published:** 2022-08-23

**Authors:** Thomas R. Porter, Steven B. Feinstein, Roxy Senior, Sharon L. Mulvagh, Petros Nihoyannopoulos, Jordan B. Strom, Wilson Mathias, Beverly Gorman, Arnaldo Rabischoffsky, Michael L. Main, Andrew Appis

**Affiliations:** 1 982265 Nebraska Medical Center, Omaha, NE 68198-2265 USA; 2 1653 W. Congress Pkwy, Chicago, IL 60612 USA; 3 Royal Brompton Hospital and Imperial College, London, UK; 4 Queen Elizabeth II Health Sciences Center, Halifax Infirmary Site, 1796 Summer Street, Room 2148.5, Halifax, NS B3H 3A7 Canada; 5 Imperial College London, Hammersmith Campus, 72 Du Cane Rd, London, W12 0HS UK; 6 Harvard Richard A. and Susan F. Smith Center for Outcomes Research in Cardiology, 375 Longwood Avenue, 4th floor, Boston, MA 02215 USA; 7 Echocardiography Lab, Heart Institute (InCor) Clinic Hospital, The University of São Paulo, Av. Dr. Enéas de Carvalho Aguiar, 44 - Cerqueira Cesar, São Paulo, SP 05403-000 Brazil; 8 Southport, USA; 9 Pro Cardiaco and America Medical City Rj, Rua General Polidoro 192, Botafogo, Rio de Jeneiro, CEP 22640-150 Brazil; 10 Saint Luke’s Mid America Heart Institute, 901 E. 104th Street, Kansas City, MO 64131 USA; 11 Kaiser Permanente Medical Center, 4647 Zion Ave, San Diego, CA 92120 USA

**Keywords:** Contrast echo, Contrast Echocardiography, Contrast enhanced ultrasound, CEUS, Protocols, Ultrasound enhancing agent, UEA, Ultrasound contrast agent

## Abstract

The present CEUS Cardiac Exam Protocols represent the first effort to promulgate a standard set of protocols for optimal administration of ultrasound enhancing agents (UEAs) in echocardiography, based on more than two decades of experience in the use of UEAs for cardiac imaging. The protocols reflect current clinical CEUS practice in many modern echocardiography laboratories throughout the world. Specific attention is given to preparation and dosing of three UEAs that have been approved by the United States Food and Drug Administration (FDA) and additional regulatory bodies in Europe, the Americas and Asia–Pacific. Consistent with professional society guidelines (J Am Soc Echocardiogr 31:241–274, 2018; J Am Soc Echocardiogr 27:797–810, 2014; Eur Heart J Cardiovasc Imaging 18:1205, 2017), these protocols cover unapproved “off-label” uses of UEAs—including stress echocardiography and myocardial perfusion imaging—in addition to approved uses. Accordingly, these protocols may differ from information provided in product labels, which are generally based on studies performed prior to product approval and may not always reflect state of the art clinical practice or guidelines.

## Introduction

Contrast-enhanced ultrasound (CEUS) is a safe, reliable, cost-effective, and reliable non-invasive imaging tool that is used worldwide to detect heart disease by enhancing left ventricular opacification, augmenting Doppler signals, and assessing myocardial perfusion [1–3]. During a CEUS exam, an ultrasound enhancing agent (UEA)^1^ is administered intravenously in a slow bolus or as a continuous infusion. UEAs are comprised of suspensions of gas-filled echogenic microspheres (sometimes referred to as “microbubbles”) with a phospholipid or albumin shell. They are smaller than red blood cells and flow throughout the patient’s microcirculation at physiologic transit times, effectively functioning as surrogate markers of perfusion. CEUS exams often reduce the need for additional more expensive downstream tests, provide results in real time and without delays often associated with accessing alternative imaging options, increase the efficiency of hospital workflows, and improve patient outcomes and experiences [4, 5]. CEUS does not expose patients or staff to ionizing radiation, and UEAs present no known risk of nephrotoxicity. In addition, because of their rapid metabolism, UEAs do not deposit or accumulate in tissues such as the brain. In critically ill patients, the use of UEAs has been associated with lower mortality [6–8]. For these reasons, UEAs are an essential component of echocardiography laboratories throughout the world.

## Objective of contrast echo study

UEAs are used to provide the highest quality ultrasound images of the cardiac anatomy and function. Real-time CEUS images help detect and stratify risk of cardiovascular disease by:

- Improving endocardial border delineation and regional wall motion analysis for the detection of coronary artery disease and cardiomyopathies;
- Enhancing Doppler signals for the determination of pulmonary artery pressure and valve gradients;
- Generating left ventricular opacification to quantify left ventricular volumes and ejection fraction;
- Generating cardiac chamber and vascular opacification to confirm or exclude intracardiac thrombi and masses; and assist in evaluating aneurysms, dissections, and carotid artery disease.
- Demonstrating microvascular perfusion.

These applications apply to both rest and stress echocardiograms.

The use and dosage of UEAs will be determined by the cardiologist supervising the procedure, or as per departmental protocol. We recommend that UEAs be administered by a physician, qualified sonographer or nurse.

## Instrumentation and settings

Most echocardiographic vendors have presets for contrast very low mechanical index (MI) imaging, which ensures the optimal pulse sequence schemes are being utilized to detect contrast. The optimal MI is the lowest MI that provides strong enhancement with sufficient penetration (absence of attenuation) and which minimizes background noise. Optimal MI will vary based on the patient and specific ultrasound machine.

Fundamental non-linear multi-pulse imaging is preferred over harmonic imaging, and a very low mechanical index (VLMI) of < 0.2 will reduce destruction of the microspheres and improve contrast when compared to harmonic imaging. With more recent industry platform changes, the optimal MI may be even lower at 0.10–0.14. Since the fundamental non-linear imaging is multi-pulse, the frame rate (temporal resolution) is less than harmonic imaging.

The transmit focus may require adjustment, but in nearly all circumstances should be positioned at the mitral annulus to reduce attenuation in the far field and visualize leaflet insertion site for endocardial tracing required for LV volumes and EF measurements. In certain circumstances, the focus can be moved to the near field to better visualize apical thrombus, morphology, wall motion, or perfusion.

The optimal transmit frequency for fundamental non-linear imaging should be 1.6–2.0 Megahertz (MHz), while for harmonic imaging 1.3–1.5 MHz works well in most adults (2.6–3.0 MHz receive frequency). In general, higher frequencies provide less penetration but superior image resolution, and large patients may require relatively higher MI and/or lower frequency.

## General principles: dosing, administration

Optimal dosing often represents a compromise between attenuation (indicating the dose is too high) and duration (which may be too short if the dose is too low). The speed of injection/flushing may also impact the quality of the CEUS image; faster injection or infusion rates may create higher concentrations of microbubbles in the apex, resulting in basal attenuation artifact, whereas slower injection rates may result in swirling artifact and incomplete LV opacification.

Bolus administration generally reduces imaging and preparation time but, due to higher UEA concentrations, attenuation artifacts may be seen. Very low UEA doses with slow saline flushes (3–5 ml slow normal saline flush over a period of at least 5 s) are recommended. Repeat injections may be needed to acquire all image planes, when bolus administration is used. Alternatively, continuous infusions provide more prolonged and consistent enhancement with less attenuation artifacts, and may allow for individual optimization of contrast enhancement by adjusting the infusion rate during imaging. While both bolus injections and continuous infusions can be used to evaluate myocardial perfusion, continuous infusions permit better quantification of myocardial blood flow abnormalities with a high MI “flash” followed by a very low MI replenishment scheme.

20 G needles or larger are recommended because the UEA may be degraded by a small caliber intravenous cannula. Similarly, a small lumen tubing should be avoided because it may destroy microbubbles even if a large needle is used. Cannulation of an antecubital or central rather than a hand vein is preferred because the UEA delivery site is into a larger vessel nearer to the heart. The use of a 3-way stopcock with contrast connected to the straight line and saline connected to the T-port may allow for the safe change of syringes.

See prescribing information for additional details.

## Preparation and administration of UEAs

Three UEAs are available commercially for enhancement of echocardiograms: Definity/Luminity (Lantheus Medical Imaging, North Billerica MA); Lumason/Sonovue (Bracco Imaging, Milan, Italy); and Optison (GE Healthcare, Chicago, Illinois). These agents may be administered via bolus injection or continuous infusion. Preparation and administration of each UEA differs, and prescribing information is available on the ICUS website: http://icus-society.org/resources/product-labels/.

Definity/Luminity is activated by agitating the vial for 45 s in a VIALMIX or VIALMIX RFID device, and then withdrawn from the vented vial. Before activating Definity/Luminity, the refrigerated vial should warm to room temperature. The product may be used for up to 12 h at room temperature after activation, but if not used within 5 min the activated vial should be resuspended by gentle hand agitation for 10 s prior to use. Intermittent slow agitation is recommended to maintain microbubble suspension. The recommended bolus dose is 0.1 ml, with a slow (10 s) normal saline flush. Higher doses can be given if needed to achieve adequate homogenous left ventricular opacification. Definity/Luminity can also be diluted in 10 to 60 ml of saline solution and proportionally larger (more easily delivered) injections (0.5–1.0 ml) may be administered, followed by the same flushing technique. Alternatively, Definity/Luminity can be given as an approximate 3–5% infusion in saline to provide more consistent opacification.

Lumason/Sonovue is activated by assembling a plunger and prefilled 0.9% saline injection syringe barrel, and connecting the syringe to a provided mini-spike that is inserted into the rubber stopper of the vial. The mini-spike contains a sterile filter and allows protected ventilation of the vial. The saline is then emptied into the vial by pushing on the plunger rod and shaking vigorously for 20 s, producing a homogeneous white milky liquid. In Europe, VUEJECT, a custom-designed infusion pump that continuously oscillates to agitate the agent, is available to maintain homogenous suspension after infusion. (The VUEJECT pump is not currently available in North America.) The VUEJECT pump continues to oscillate after initial mixing is complete. The infusion rate can be controlled via a touchpad and increased or decreased as necessary. Lumason/Sonovue may be used for up to 3 h after reconstitution; in the event of a delay, the microspheres should be resuspended for a few seconds by hand agitation before the suspension is drawn into the syringe. If given as a bolus, the recommended initial dose for adults should be 0.5 ml followed by a slow five milliliter saline flush over 10 s. The dose can be increased to 1.0 ml to achieve homogenous left ventricular opacification. The optimal dose should be repeated every 30 s to maintain opacification. The recommended initial dose for pediatric patients is 0.03 ml/kg.

Optison vials should be inverted and gently rotated to resuspend the microspheres; this process will allow the product to come to room temperature before use. The Optison suspension will be homogeneous, opaque, and milky-white. The vial of suspended Optison should be vented with a sterile vent spike or with a sterile 18 gauge needle before the solution is withdrawn into the injection syringe. The product should be used within 1 min of suspension, and in the event of a delay the product should be resuspended by inverting and gently rotating the microspheres in the syringe. (Some clinicians have successfully used an infusion pump or Dial-A-Flow with Optison, typically 1.5 ml in no more than 20 ml of saline). The IV line should be shaken gently and tapped to prevent Optison microspheres from adhering to the sides or separating. The recommended initial bolus dose of Optison is 0.3–0.5 ml followed by a slow 5 ml normal saline flush over 10 s.

## Protocol: bolus administration during rest or stress echocardiogram

1. Suggested initial doses for each agent are provided above. We recommend small doses for resting or stress echocardiograms.
  When using VLMI fundamental non-linear imaging, higher doses are rarely necessary. Always start with lower doses to avoid shadowing/attenuation and waste of the UEA. Dosing may be adjusted according to adequacy of visualization of the UEA in the cardiac chambers.
2. Use a very slow saline flush via syringe (approximately 5 ml over 10 s). Stop flushing when the UEA appears in the right ventricle. Alternatively, use a saline drip, adjusting the rate as required to obtain a good image.
3. To prolong the contrast effect, a second dose may be administered, followed by a second saline flush. For most applications, this is typically 30 s after the first dose, but will vary from patient to patient.
4. For pharmacologic stress studies, wall motion images should be acquired at baseline, low dose, and peak stress; additional imaging may be done at pre-peak, and in recovery.
5. For exercise stress studies, the UEA bolus and saline flush should be administered at rest, and then again approximately 15–30 s before termination of exercise, depending in part upon the UEA being administered. For pharmacologic stress studies, the UEA bolus and flush can be administered at rest, and during the infusion through the same IV line as the pharmacological stressor; in general, a flush is not needed when administering in the same line as the stress agent infusion.
6. Avoid flushing vigorously or applying strong pressure to the infusion or injection because doing so may destroy the UEA microspheres.
7. Set up the ultrasound system to utilize contrast presets.
  VLMI (< 0.2) fundamental non-linear multi-pulse imaging, such as amplitude modulation or combined amplitude/phase modulation, is preferred for BOTH optimal left ventricular opacification (LVO) and perfusion imaging.
  Alternatively, second harmonic low mechanical index (LMI) (0.2–0.3) imaging may be used, but is less optimal and will achieve only LVO.
  Caution: Reducing MI in the LVO mode may not achieve the image quality of the dedicated VLMI contrast preset, as the signal to noise ratio is significantly reduced when MI < 0.25. However, recent industry platform changes have included fundamental non-linear imaging as an option for the LVO setting, with the same multi-pulse sequence used for perfusion imaging but with slightly different dynamic range and filtering. This permits taking advantage of a dedicated VLMI setting to improve the signal to noise ratio for LVO and endocardial border delineation of all segments at an MI < 0.20.
8. Acquire images when:
  For LVO: Homogenous left ventricular cavity contrast enhancement without swirling or blooming in the apex or shadowing/attenuation in the basal segments is seen. Contrast should be visible in the left atrium approximately 1–2 cm behind the mitral valve.
    As previously described, this is best achieved with a fundamental non-linear multi-pulse imaging technique such as amplitude modulation or combined amplitude/phase modulation. Near and far field time gain compensation adjustments may be needed, and transmit focus may need to be adjusted. Repeat UEA bolus and flush as needed to maintain optimal visualization.
  For perfusion: Proceed as above, with additional step of using high mechanical index (high MI) “flash” impulse, which will destroy the UEA within the myocardium, without reduction in LV opacification. The high MI flash frame number and/or flash frame mechanical index can be adjusted to achieve UEA destruction within the myocardium without causing loss of microsphere signal in the LV cavity. With current two-dimensional transducers, normal myocardial perfusion is indicated by complete replenishment of myocardial contrast within 5 s of the high mechanical index (MI) impulse under resting conditions, and within 2 s under stress conditions. Abnormal myocardial perfusion is indicated by delayed or absent replenishment after flash high MI impulses, and may be subendocardial or transmural in distribution.
    Myocardial perfusion consists of two elements: blood volume and blood flow velocity. The absence of blood volume at peak enhancement can indicate the presence of a myocardial infarction or severely reduced microvascular perfusion. In addition, delayed replenishment of blood flow following microsphere destruction occurs due to reduced myocardial blood flow and can indicate a hemodynamically significant epicardial coronary stenosis and/or compromised microcirculation.
    Myocardial perfusion imaging should be combined with wall motion analysis. During stress testing, perfusion imaging is more sensitive and may rule-in high-risk patients, whereas wall motion analysis is more specific and may rule-out low-risk patients.

## Protocol: continuous infusion during rest or stress echocardiogram

The following protocol should be used for continuous infusion of the UEA:

1. Make 2 syringes—One for REST, one for STRESS (if stress test is utilized). Use ½ vial of contrast for each setting and dilute in 20–30 ml of normal saline if you are utilizing a continuous infusion.
2. Adjust dose to achieve the following image appearance of the left ventricle from the apical windows:
  For LVO: Homogenous left ventricular cavity contrast without swirling in the apex or shadowing of basal segments. This is best achieved with a fundamental non-linear multi-pulse imaging technique such as amplitude modulation or combined amplitude/phase modulation. Mechanical index should be < 0.2 for fundamental non-linear imaging and 0.25–0.30 for harmonic imaging.
  For LVO with perfusion: homogenous myocardial contrast opacification in all segments with normal wall thickening (using near and far field time gain compensation adjustments), and adjustment of transmit focus.
  Complete disappearance of myocardial contrast following high mechanical index “flash” impulses (0.8–1.2 mechanical index (MI), 5–20 frames) with minimal evidence of reduced LV opacification [achieved by adjusting the flash frame number and/or flash frame mechanical index (MI)].
  Normal myocardial perfusion: near-complete replenishment of myocardial contrast within 5 s of the high mechanical index impulse under resting conditions, and within 2 s under any type of stress (exercise, dobutamine or vasodilator), is consistent with normal perfusion.
  Abnormal myocardial perfusion: findings other than this would be considered abnormal perfusion. Attenuation must be ruled out if any delay is confined to just basal segments in apical windows. The apical windows can be purposefully foreshortened to bring these basal segments into the near field for better evaluation should attenuation be suspected. In addition, the image plane can be adjusted to bring the myocardial segments of interest nearer to the center of the image, rather than at the sides of the imaging sector where contrast sensitivity is lower. Near field time gain compensation should be adjusted higher for most systems to avoid the appearance of decreased apical segment contrast enhancement. More recent platforms have built in this adjustment in the near field and no near field upward adjustment in time gain compensation is necessary. This should be optimized under resting conditions.
3. Imaging mode. A fundamental non-linear setting is recommended for both LVO and myocardial perfusion imaging, if available. For both fundamental and harmonic imaging, gain settings should be adjusted and set to provide the most homogenous discernable difference between the left ventricular cavity and endocardial border of the myocardium. This varies from system to system, but should be adjusted so that a high MI reduces the visible myocardial contrast in all segments of the myocardium.
  Use both overall gain settings and slight position changes in near and far field time gain compensation potentiometers to achieve this objective.

## Storage

UEAs should be stored in a convenient location in or near the echocardiography laboratory or intensive care unit to provide for immediate access. A dedicated medical-grade refrigerator should be provided for agents requiring refrigeration.

## Precautions

Serious adverse reactions to UEAs are rare and occur in approximately 1 in 10,000 patients [9, 10]. Prior to administering UEAs, it should be established that the patient has no known or suspected hypersensitivity to the components of the specific UEA. In addition, epinephrine, diphenhydramine and/or steroids should be available to treat a rare adverse event associated with hypersensitivity to UEAs, and staff should be trained to promptly recognize and treat any adverse reaction.

## UEA product labels: global

For current product labels approved in jurisdictions around the world, please visit the ICUS website: http://icus-society.org/resources/product-labels/.

## Disclaimer

The information and protocols contained in this document do not constitute the offering of medical advice, nor do they create a physician–patient relationship between the International Contrast Ultrasound Society (ICUS) or its directors or members, and any other party. ICUS makes no express or implied warranties regarding the accuracy or completeness of the information and protocols contained in this document, and ICUS shall not be liable to any party with respect to any medical decision made, or any action taken or not taken, in direct or indirect reliance on the information and protocols contained in this document.

## Data Availability

Not applicable.

## Footnotes

1 Ultrasound enhancing agents are also sometimes known as ultrasound contrast agents.

## References

[1] Porter TR, Mulvagh SL, Abdelmoneim SS, Becher H, Belcik JT, Bierig M (2018). Clinical applications of ultrasonic enhancing agents in echocardiography: 2018 American society of echocardiography guidelines update. J Am Soc Echocardiogr.

[2] Porter TR, Abdelmoneim S, Belcik JT, McCulloch ML, Mulvagh SL, Olson JJ (2014). Guidelines for the cardiac sonographer in the performance of contrast echocardiography: a focused update from the American society of echocardiography. J Am Soc Echocardiogr.

[3] Senior R, Becher H, Monaghan M, Agati L, Zamorano J, Vanoverschelde J, Nihoyannopoulos P, Edvardsen T, Lancellotti P (2017). Clinical practice of contrast echocardiography: recommendation by the European association of cardiovascular imaging (EACVI). Eur Heart J Cardiovasc Imaging.

[4] Kurt M (2009). Impact of contrast echocardiography on evaluation of ventricular function and clinical management in a large prospective cohort. J Am Coll Cardiol.

[5] Streb JW (2019). Retrospective analysis of contrast-enhanced ultrasonography effectiveness in reducing time to diagnosis and imaging-related expenditures at a single large United States County Hospital. Ultrasound Q.

[6] Main ML, Ryan AC, Davis TE (2008). Acute mortality in hospitalized patients undergoing echocardiography with and without an ultrasound contrast agent (multicenter registry results in 4,300,966 consecutive patients). Am J Cardiol.

[7] Kusnetzky LL, Khalid A, Khumri TM (2008). Acute mortality in hospitalized patients undergoing echocardiography with and without an ultrasound contrast agent: results in 18,671 consecutive studies. J Am Coll Cardiol.

[8] Main ML, Hibberd MG, Ryan A (2014). Acute mortality in critically ill patients undergoing echocardiography with or without an ultrasound contrast agent. JACC Cardiovasc Imaging.

[9] Wei K, Mulvagh SL, Carson L, Davidoff R, Gabriel R, Grimm RA (2008). The safety of Definity and Optison for ultrasound image enhancement: a retrospective analysis of 78,383 administered contrast doses. J Am Soc Echocardiogr.

[10] Muskula PR, Main ML (2017). Safety with echocardiographic contrast agents. Circ Cardiovasc Imaging.

